# High Expression of Tumor Abnormal Protein Preoperatively Predicts Poor Prognosis of Patients With Esophageal Squamous Cell Carcinoma

**DOI:** 10.3389/fsurg.2021.609719

**Published:** 2021-02-24

**Authors:** Yuanjun Cheng, Qianru Fang, Yongbing Chen, Guohui Zang, Jie Yao

**Affiliations:** ^1^Department of Cardiothoracic Surgery, People's Hospital of Chizhou, Chizhou, China; ^2^Department of Obstetrics, People's Hospital of Chizhou, Chizhou, China; ^3^Department of Thoracic Surgery, The Second Affiliated Hospital of Soochow University, Suzhou, China

**Keywords:** tumor abnormal protein, TAP, esophageal squamous cell carcinoma, prognosis, poor

## Abstract

**Background:** Esophageal squamous cell carcinoma (ESCC) acts as a fatal malignant tumor among human beings and is marked by late-stage diagnosis, frequent recurrence, metastasis, and therapy resistance. Tumor abnormal protein (TAP) remarkably affects cancer development and progression of human cancers. TAP has been shown to be a biomarker for gastric and lung cancer progression. Nevertheless, the clinical value exhibited by TAP for ESCC has not been well-explained in the current literature.

**Methods:** The present study included 183 ESCC cases who received surgical resection and 183 cases who had normal physical checkup from March 2013 to January 2015 at the People's Hospital of Chizhou, and used the TAP detection agent for evaluating the TAP relative level.

**Results:** As found, ESCC patients presented an obviously higher TAP expression relative to cases who had normal physical checkup. Moreover, TAP expression was significantly downregulated after surgery. Furthermore, the TAP expression was correlated with gender, smoking, pathologic differentiation, and pN stage, but not with age, tumor location, surgical type, pT stage, and vascular invasion. High expression of TAP was significantly correlated with poorer overall survival (OS) rate in ESCC patients. TAP was an independent prognostic predictor in ESCC patients, based on the multivariate survival analysis.

**Conclusion:** The study reveals how TAP upregulation promotes ESCC malignant progression, and concludes that TAP acts as the therapeutic target and potential biomarker specific to ESCC.

## Introduction

Up to now, esophageal cancer ranks seventh among all common cancer types and ranks sixth among all the causes that lead to cancer-related deaths all over the world. Its 5-year survival rate is <20% (1). Esophageal squamous cell carcinoma (ESCC) is a prevalent type of esophageal cancer (occupying over 90%) worldwide, with a high incidence in Asia, South America, and East Africa (2). Despite the oncology development as well as multidisciplinary treatment, its recurrence and mortality remain high. One important reason is that most ESCC patients present with advanced stage at the time of diagnosis. Therefore, there is a critical need to find effective pathways by which to predict tumor genesis and development. Recently, various proteins have been found to be closely correlated with the occurrence and progression of ESCC. However, new molecules which possess diagnostic value for clinical application are still needed to be discovered. USSR scholars Kostyantin, A. and Galakhin identified the tumor abnormal protein (TAP), and many literatures revealed that TAP was closely associated with the progression and occurrence of numerous cancers (3, 4). In the process of metabolism, cancer cells are capable of emitting complicated abnormal glycoproteins as well as calcium-histone proteins which constitute TAP (5). In essence, TAP results from the glycosylation changes regarding cancer cells. It indirectly reflects the cell cancerization number and degree. Once these substances reach a given volume, they will enter into the blood and, in a larger number, remain in the peripheral blood. During the early detection stage of cancer, an increase in TAP expression is considered as a significant indicator. Thus, it is necessary to further investigate the biological action related to TAP. TAP is produced by gene (oncogene and tumor suppressor gene) mutation in cells. Besides, upregulated TAP promotes tumor growth. In various tumor types, like breast, ovarian, colon, endometrial, stomach, lung cancers, etc., the upregulation of TAP is seen (6). In addition, TAP remarkably affects tumor development, progression, and metastasis, making it a significant indicator of tumor prognosis. However, the role of TAP in the tumorigenesis and progression of ESCC is not yet clear and warrants elucidation.

The study aims at evaluating the TAP expression of ESCC patients compared with cases who had normal physical checkup. In addition, we also analyze the correlation between the TAP expression and the baseline characteristics of ESCC patients, including age, sex, smoking, tumor location, surgical type, pathologic differentiation, pT stage, pN stage, vascular invasion, and overall survival.

## Materials and Methods

### Patient and Sample Collection

The study has obtained the approval of the Ethical Committee of People's Hospital of Chizhou in Anhui Province, China, as well as received all of the participants' written informed consent forms. Experimental implementation was in accordance with the Declaration of Helsinki. Healthy patients were included as the control group. ESCC patients and cases who had normal physical checkup were also included in the study, and blood samples were collected from those who received surgical resection at the People's Hospital of Chizhou during March 2013 and January 2015. The included ESCC patients were those who did not receive radiotherapy and/or chemotherapy prior to the study. The ESCC results were evaluated histopathologically (eighth edition of the TNM Classification for Esophageal Cancer). All 183 ESCC patients' follow-up data were collected as well as retained. OS refers to the period from the time of diagnosis to the date of death or the last known date of life. Table 1 summarizes all the baseline characteristics.

**Table 1:** Clinical association of TAP expression with baseline variables of esophageal squamous cell carcinoma (ESCC) patients.

**Variable**	**Number**	**TAP**	***P*-value**	
		**Low expression**	**High expression**	
**Age**				0.069
≤ 65	102	68	34	
>65	81	43	38	
**Sex**				**0.001**
Male	97	48	49	
Female	86	63	23	
**Smoking**				**0.000**
No	62	21	41	
Yes	121	90	31	
**Tumor location**				0.987
Lower	65	37	28	
Middle	104	68	36	
Upper	8	14	6	
**Surgical type**				0.258
Sweet esophagectomy	65	37	28	
Ivor-Lewis esophagectomy	44	25	19	
Mckeown esophagectomy	74	49	25	
**Pathologic differentiation**				**0.001**
Well	31	19	7	
Moderate	98	67	31	
Poor	54	25	34	
**pT stage**				
T1	20	16	4	0.418
T2	69	38	31	
T3	82	50	32	
T4	12	7	5	
**pN stage**				**0.001**
N0	104	72	32	
N1	54	30	24	
N2	25	9	16	
**Vascular invasion**				0.844
No	159	96	63	
Yes	24	15	9	

*Bold values indicate P < 0.05*.

### TAP Detection

#### Detection Methods

Fasting blood (2 ml) was collected from the patient's fingertips in the morning. Blood smear with uniform thickness was prepared and then allowed to dry at room temperature for 10 min. Coagulants were added to the blood smear and the particles were condensed after 1.5–2 h. All blood samples were examined with the TAP reagent (Biosharp Biotech, Hefei, China), and then we searched and measured the condensed particulate matter by the TAP detection image analyzer. The TAP results of patients with esophageal cancer were examined on admission and on the first day after surgery. Routine tests were performed on the physical examination group.

#### Determination of TAP Detection Results

**Figure 1:**
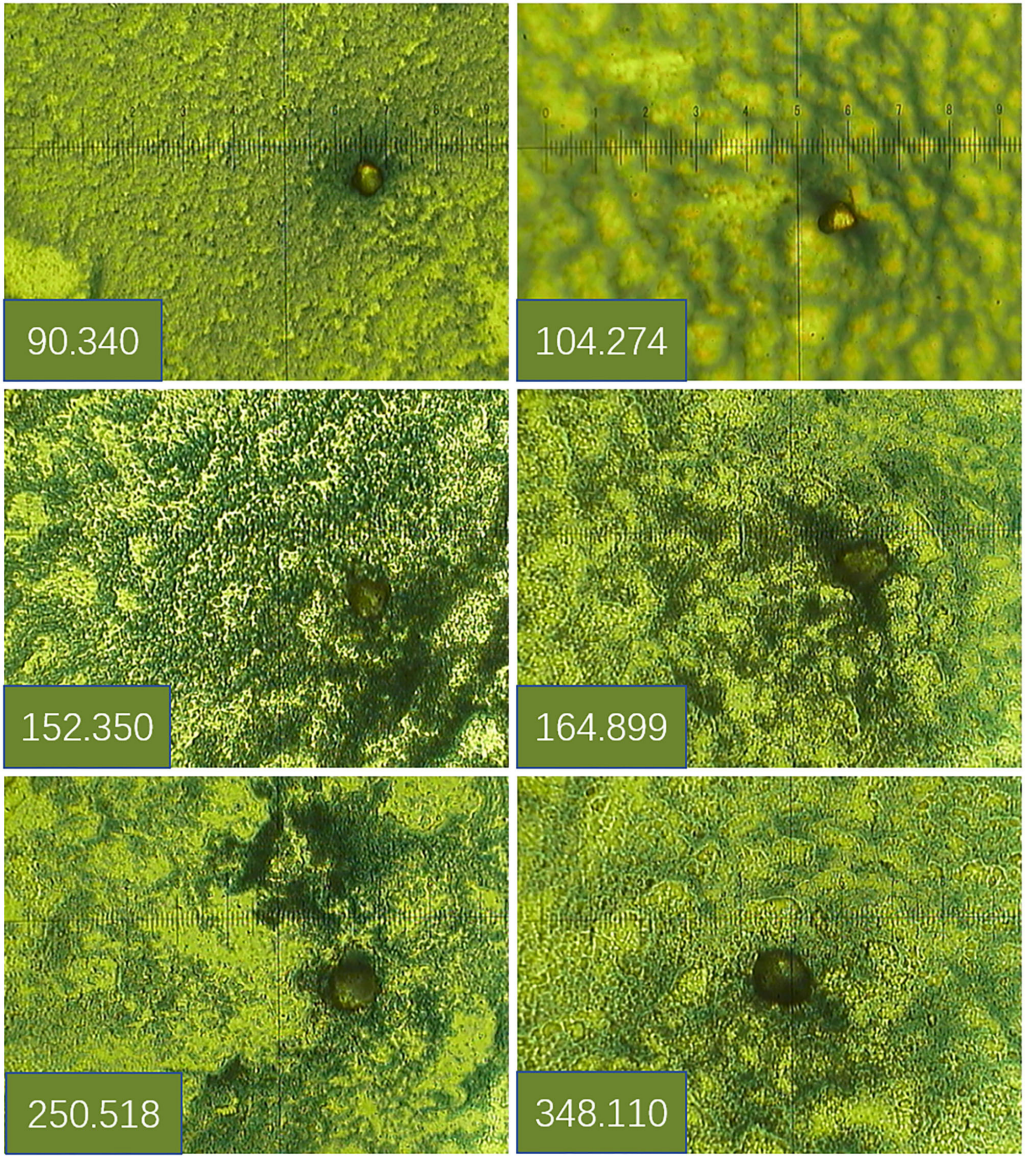
Determination of tumor abnormal protein (TAP) detection results.

TAP in the blood reacted with the reagent for generating a crystal-like condensation product, thereby proving its existence. As shown in Figure 1, TAP negative: condensate area is ≤ 121 μm^2^; TAP weakly positive or critical type: condensate area is 121–225 μm^2^; and TAP positive: the condensate area was observed as follows—the group with a high expression exhibited a condensation particle area ≥225 μm^2^, and the group with a low expression exhibited a condensation particle area <225 μm^2^.

### Statistical Analysis

Experiments were repeated for no <3 times. All statistical data were in the form of the mean ± standard error of the mean (SEM). The SPSS 23.0 software package (SPSS, Chicago, IL, USA) was applied for statistical analysis. The TAP expression levels were classified as low expression (the condensation particle area was <225 μm^2^) or high expression (the condensation particle area was ≥225 μm^2^). Independent-samples *t*-test was applied to compare two groups in terms of the TAP expression. The chi-square test was employed to evaluate the correlation of TAP expression with ESCC baseline parameters. The Kaplan–Meier method together with the log-rank test was adopted for checking and comparing the prognosis. Finally, analytical tools of univariate and multivariate analyses were employed to reveal the factors that can independently predict the prognosis of ESCC patients. *P* < 0.05 is considered exhibiting statistical significance.

## Results

### TAP Upregulation in ESCC Tissues

**Figure 2:**
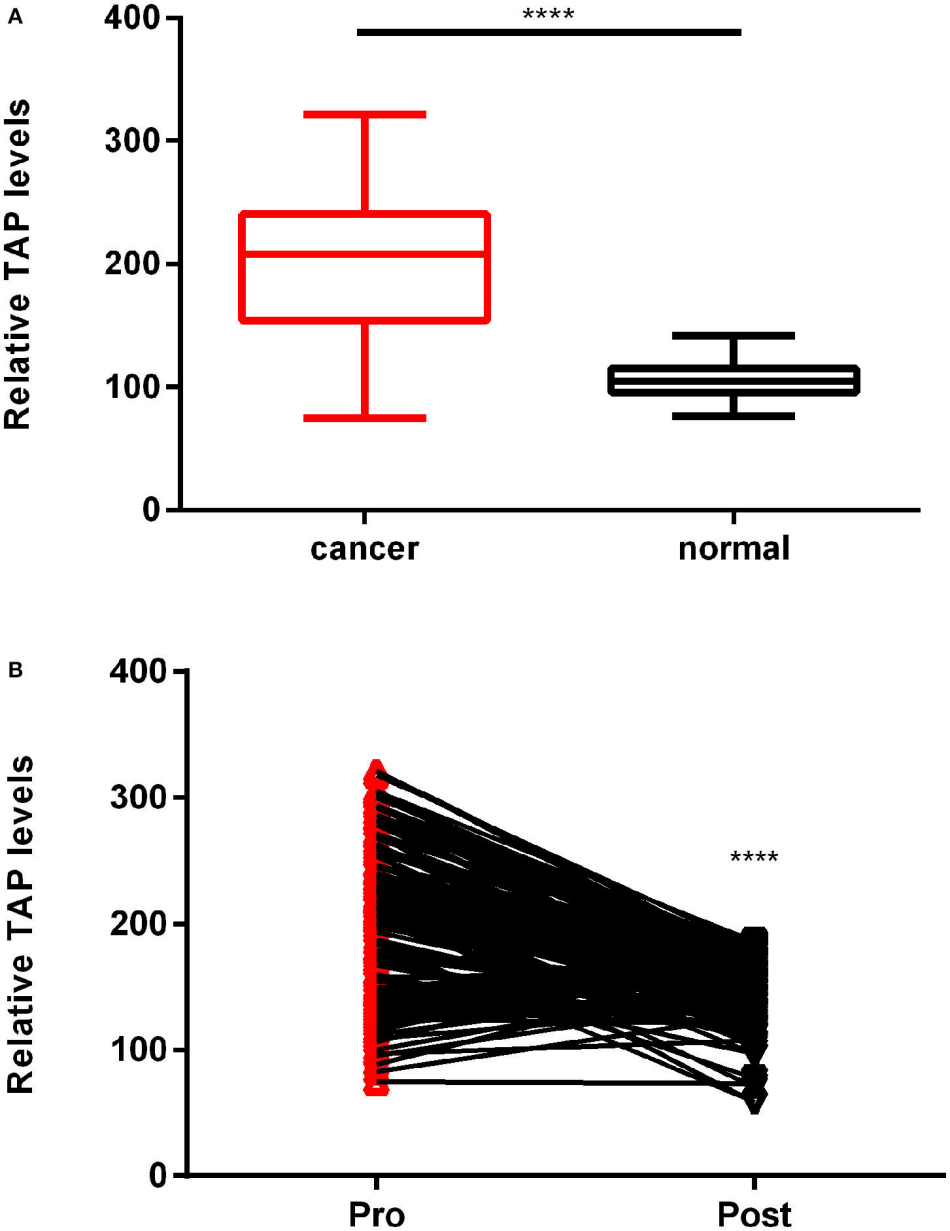
TAP relative expression in esophageal squamous cell carcinoma (ESCC) patients and cases who had normal physical checkup. **(A)** The TAP detection reagent was employed to measure the TAP expression in 183 ESCC and healthy blood samples. **(B)** A line links the pre-operation point to the post-operation point with a downward trend, demonstrating TAP downregulation in ESCC patients following surgery (^****^p < 0.0001).

In this study, we compared the expression of TAP in 183 cases of ESCC tissues before surgery and cases who had normal physical checkup. The results showed that TAP was significantly increased in ESCC tissues compared with cases who had normal physical checkup (Figure 2A). We further examined its expression in ESCC patients after surgery. As shown in Figure 2B, the results indicated that the expression level of TAP was decreased in ESCC patients after surgery. Therefore, TAP is upregulated in ESCC tissues.

### TAP Expression Is Correlated With the Patient's Sex, Smoking, Pathologic Differentiation, and pN Stage of ESCC

The mean value was taken as a standard to classify ESCC blood samples into two groups as mentioned above. Table 1 reveals that high TAP expression is closely associated with baseline factors like sex, smoking, pathologic differentiation, and pN stage of ESCC, while it is not affected by patient's age, tumor location, surgical type, pT stage, or vascular invasion. Taken together, the increase in TAP expression promotes the growth of ESCC. The association of TAP expression with ESCC baseline characteristics was evaluated to better explain the function possessed by TAP in ESCC. The TAP expression levels in ESCC tissues were categorized as two groups as mentioned above. As indicated in Table 1, high TAP expression was significantly correlated with sex, smoking, pathologic differentiation, and pN stage, whereas we did not find a correlation between TAP expression and age, tumor location, surgical type, pT stage, or vascular invasion. Taken together, the increase in TAP expression promotes the malignant progression of ESCC. The baseline data characteristics of the control group are shown in Table 2, including gender, age, and smoking.

**Table 2:** Baseline demographic data for the control group.

**Variable**	**Number**	**TAP (mean ± SD)**	***P*-value**
**Age**			0.424
≤ 65	170	104.979 ± 13.155	
>65	13	108.015 ± 13.337	
**Sex**			0.838
Male	107	105.026 ± 13.469	
Female	76	105.432 ± 12.782	
**Smoking**			0.739
No	102	104.0981 ± 13.42264	
Yes	81	106.5760 ± 12.75596	

### For ESCC Patients, High TAP Expression Indicates Poor Prognosis

**Figure 3:**
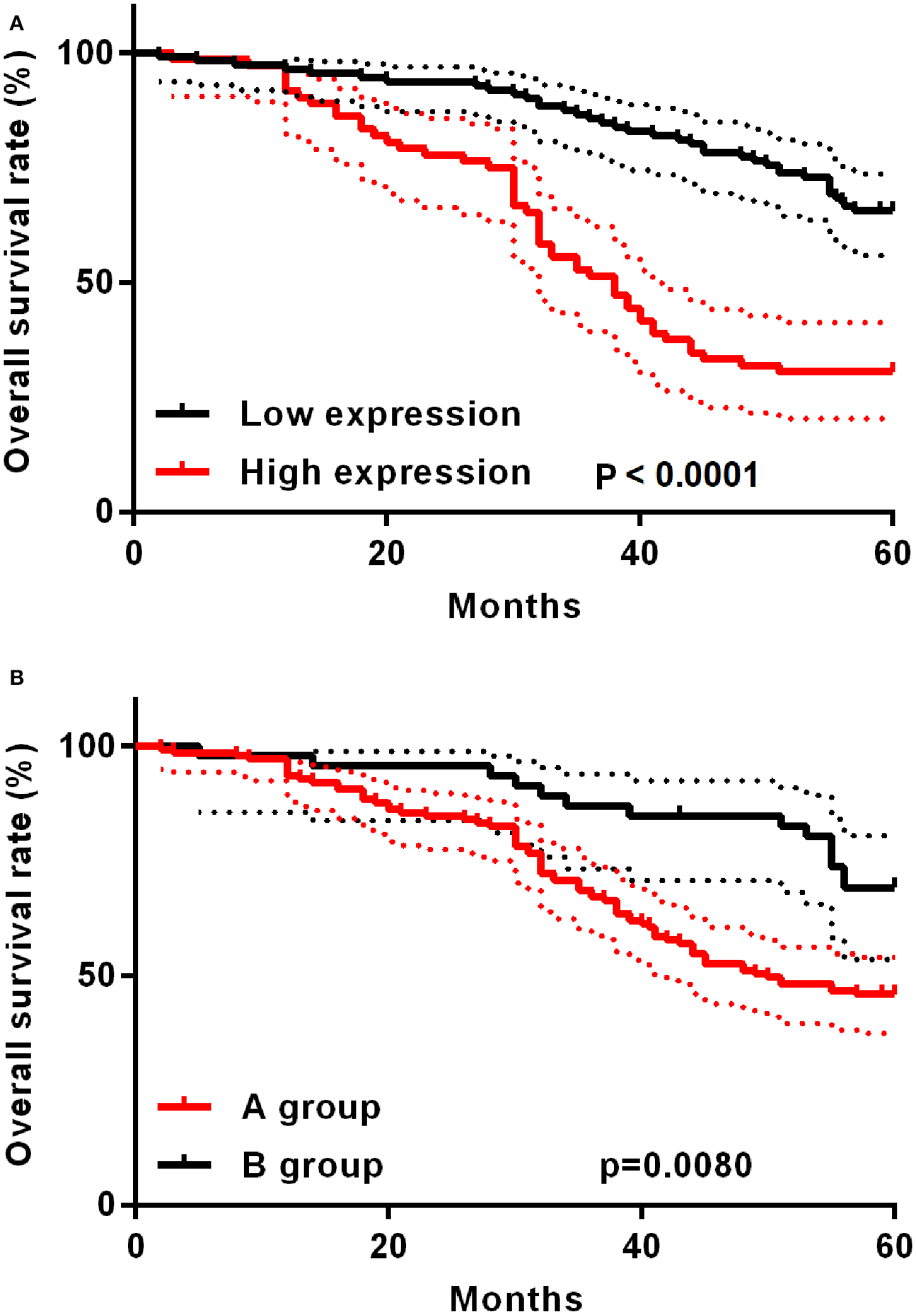
Kaplan–Meier post-operative survival curve specific to ESCC patients with TAP expression. **(A)** For ESCC patients whose TAP expression is high (*n* = 72), the survival time is shorter relative to those whose TAP expression is low (*n* = 111). **(B)** The A group prognosis is worse than the B group. A group: post-operative TAP/pre-operative TAP ratio <1; B group: post-operative TAP/pre-operative TAP ratio >1.

The study deeply analyzed as well as evaluated the correlation between TAP expression and the survival time of ESCC patients, finding that high TAP expression led to weaker prognosis relative to low TAP expression (Figure 3A). After subgroup analysis, it was found that patients with higher post-operative than pre-operative levels had better prognosis than patients with lower post-operative levels (Figure 3B). The analytical tools of univariate and multivariate Cox proportional hazards assisted in studying independent factors that predicted survival in ESCC patients. As revealed by univariate analysis data, the overall survival in ESCC patients was significantly correlated with TAP expression, pathologic differentiation, pT stage, and pN stage (Table 3). In addition, TAP expression is also an independent prognostic factor for ESCC patients (Table 4), whereas pathologic differentiation and pN stage were not independent prognostic factors affecting the overall survival of ESCC patients (Table 4). Therefore, our data suggests that high expression of TAP can predict poor prognosis in ESCC patients.

**Table 3:** Univariate analysis on ESCC prognostic factors.

**Variable**	**HR(95% CI)**	***p*-value**
TAP expression (Low/High)	3.051 (2.017–4.615)	**0.000**
Age (≤ 65/>65)	0.762 (0.505–1.151)	0.197
Sex (Male/Female)	0.844 (0.562–1.267)	0.413
Smoking (Yes/No)	1.493 (0.986–2.260)	0.058
Tumor location (Lower/middle/upper)	1.074 (0.757–1.524)	0.688
Surgical type (Sweet/Ivor-Lewis/Mckeown)	1.018 (0.803–1.289)	0.885
Pathologic differentiation (Well/moderate/poor)	1.436 (1.042–1.980)	**0.027**
pT stage (T1/T2/T3/T4)	1.414 (1.079–1.854)	**0.012**
pN stage (N0/N1/N2)	1.431 (1.088–1.882)	**0.010**
Vascular invasion (No/Yes)	1.349 (0.776–2.347)	0.288

Bold values indicate P < 0.05.

**Table 4:** Multivariate analysis on ESCC-independent prognostic factors.

**Variable**	**HR(95% CI)**	***p*-value**
TAP expression (Low/high)	3.055 (1.964–4.751)	**0.000**
Pathologic differentiation (Well/moderate/poor)	1.066 (0.766–1.484)	0.705
pT stage (T1/T2/T3/T4)	1.532 (1.160–2.022)	**0.003**
pN stage (N0/N1/N2)	1.197 (0.898–1.594)	0.219

*Bold values indicate P <0.05*.

## Discussion

Studies performed recently have proven that TAP remarkably affects cancer development and progression and regulates cell growth in terms of proliferation and apoptosis (7–9). Gastric carcinoma patients presented an obvious TAP upregulation relative to healthy participants. Besides, patients whose TAP expression was high showed an obvious progression-free survival (PFS) (10). TAP expression in urothelium carcinoma cells of the bladder was detected and examined based on the symptoms and clinical signs (11). TAP detection exhibited a stronger specificity and sensitivity for colorectal cancer (CRC) patients. Meanwhile, TAP detection also served to independently indicate CRC growth during chemotherapy and clinical monitoring process (12). The unique function possessed by specific TAP shall be essentially figured out to promote the advancement of diagnosis and therapy regarding cancers.

The study adopted 183 peripheral blood samples from ESCC patients while assessing TAP expression. As revealed by TAP detection data, TAP was remarkably upregulated in ESCC tissues compared with cases who had normal physical checkup. Besides, its expression levels were also decreased in ESCC patients after surgery. Therefore, TAP may be involved in the development of ESCC. Besides, according to a thorough analysis, the high expression of TAP was significantly correlated with the patient's sex, smoking, pathologic differentiation, and pN stage of ESCC, but there was no correlation between TAP expression and age, tumor location, surgical type, pT stage, or vascular invasion of ESCC. Because high invasion and metastasis of the tumor are often responsible for poor prognosis in cancer patients, we hypothesized that TAP might affect the prognosis of ESCC patients. To prove the hypothesis, we analyzed the correlation between the TAP expression and the overall survival of ESCC patients. As confirmed, ESCC patients whose TAP expression was high exhibited a weaker prognosis, relative to those whose TAP expression was low. Also, TAP expression was an independent prognostic factor in ESCC patients. Subgroup analysis found that patients with a higher post-operative level than a pre-operative level had better outcomes than patients with a lower post-operative level. The causes were analyzed: (1) Patients with elevated post-operative expression were all patients with low pre-operative expression. (2) Patients with high pre-operative expression had vigorous tumor metabolism and the TAP secreted into the peripheral blood had reached the peak. (3) In patients with low pre-operative expression, the secreted TAP did not reach the peak, and the tumor activity increased after surgical stimulation, leading to an increase in post-operative TAP.

Furthermore, breast cancer patients presented a higher level of TAP expression relative to patients who had a benign diagnosis (*P* < 0.001). There was no correlation between TAP and tumor size, estrogen and progesterone receptors, and her-2 expression, as well as pathological degree (13). By contrast, TAP could be remarkably affected by the patient's age, lymph node metastasis, and TNM stage (13). Based on recent findings, TAP could be utilized to diagnose lung cancer as well as evaluate lung cancer progression (14). TAP detection could assist in sensitively identifying malignant tumor-related aberrant sugar chains in the digestive tract. Hence, it was possible to obtain many tumor-related signals. TAP could be detected in malignant tumors in subclinical stage (15–17). Previous studies verified that TAP detection was achieved 2 years earlier than the discovery of clinical signs and related symptoms as well as malignant lumps (18). It was suggested to further study the exact function and mechanism regarding TAP in regulating ESCC cell proliferation, migration, and invasion, which exhibited an increasing significance as TAP-positive patients showed a greater need for therapeutic interventions as well as for the prevention and treatment of cancers (19, 20).

In addition to ESCC, TAP was also obviously expressed in stomach cancer, colorectal cancer, thyroid cancer, and bladder cancer, as well as lung cancer (9–12, 21, 22). The results of the study generally reviewed the role played by TAP in the development as well as the progression of tumor.

To sum up, TAP expression is dramatically upregulated and downregulated in ESCC blood before and after surgery, respectively. Moreover, TAP expression obviously relates to patient's sex, smoking, pathologic differentiation, and pN stage. Besides, the increase in TAP expression can better predict the weaker ESCC prognosis. Taken together, the study aims at expounding how TAP expression promotes ESCC malignant progression as well as serves as a biomarker for ESCC prognosis. However, this study has some limitations: The lack of benign esophageal tumors as a suitable control group reduces the scientific nature of TAP assessment, and the small sample size objectively reduces the scientific significance of TAP in the diagnosis of esophageal squamous cell carcinoma. These problems need to be further explored in subsequent studies.

## Data Availability Statement

The original contributions presented in the study are included in the article/supplementary material, further inquiries can be directed to the corresponding author/s.

## Ethics Statement

The study has obtained the approval of the Ethical Committee of ChiZhou People's Hospital in Anhui Province, China, as well as received all participants' written informed consents.

## Author Contributions

All authors listed have made a substantial, direct and intellectual contribution to the work, and approved it for publication.

## Conflict of Interest

The authors declare that the research was conducted in the absence of any commercial or financial relationships that could be construed as a potential conflict of interest.
